# PUM1 Is Overexpressed in Colon Cancer Cells With Acquired Resistance to Cetuximab

**DOI:** 10.3389/fcell.2021.696558

**Published:** 2021-08-10

**Authors:** Qizhi Liu, Cheng Xin, Yikuan Chen, Jiawen Yang, Yingying Chen, Wei Zhang, Lechi Ye

**Affiliations:** ^1^Department of Colorectal Surgery, Changhai Hospital, Shanghai, China; ^2^Department of Colorectal Surgery, Fudan University Shanghai Cancer Center, Shanghai, China; ^3^Department of Oncology, Shanghai Medical College, Fudan University, Shanghai, China; ^4^Wenzhou Medical University, Wenzhou, China; ^5^Department of Colorectal and Anal Surgery, The First Affiliated Hospital of Wenzhou Medical University, Wenzhou, China

**Keywords:** colon cancer, cetuximab, PUM1, DDX5, acquired resistance

## Abstract

**Background:**

Cetuximab is an effective antibody to treat colorectal cancer (CRC) by targeting the epidermal growth factor receptor (EGFR). However, the mechanisms of acquired resistance to cetuximab therapy, especially in patients without identifiable gene mutations, are not fully understood.

**Methods:**

Our study investigated the role of pumilio RNA-binding family member 1 (PUM1) in cetuximab resistance. We established cetuximab-resistant colon cancer cell lines SW480R and Caco-2R and knocked out PUM1 and DEAD-box helicase 5 (DDX5) with the clustered regularly interspaced short palindromic repeats (CRISPR)-caspase 9 (Cas9) system. To check cell proliferation, we used Cell Counting Kit-8. We performed qPCR and immunoblot to examine the levels of mRNAs and proteins for each cell line.

**Results:**

Our data showed that PUM1 was upregulated in SW480R and Caco-2R cells with increased protein levels and cell proliferation, and PUM1 knockout reduced cell viability in the presence of cetuximab. We also found that PUM1 interacted with DDX5 in 3′ untranslated region (UTR) and positively regulated its mRNA expression. Furthermore, suppression of DDX5 also decreased the proliferation of SW480R and Caco-2R cells.

**Conclusion:**

Our study suggests that PUM1 positively regulates DDX5 and acts as a promoter in cetuximab-resistant colon cancer cells.

## Introduction

Colon and rectal cancers occurring in the lower digestive tract were usually described as colorectal cancer (CRC), which is the third most common malignant carcinoma in the world. According to the World Health Organization, there were 1.8 million new cases of CRC in 2018, which subsequently caused 861,000 deaths (Global Burden of Disease Cancer Collaboration et al., 2019). According to the American Cancer Society, the estimated number of new cases of CRC in the United States is 147,950 in 2020, with an estimate of 53,200 cases of CRC-caused death (Siegel et al., 2014). The risk factors of CRC include environmental (such as obesity, red meat, alcohol, and diet), a history of adenomatous polyps and inflammatory bowel disease, and specific gene disorders such as inherited Lynch syndrome (MLH1, MSH2, or MSH6 gene mutations) (Keum and Giovannucci, 2019). Apart from surgical and chemotherapy treatment, targeted therapy is used to suppress specific factors that promote tumor growth in CRC. Such targets include vascular endothelial growth factor (VEGF) and epidermal growth factor receptor (EGFR), the latter of which promotes tumor cell growth (Kim and Park, 2016). Cetuximab is a chimeric immunoglobulin G1 (IgG1) monoclonal antibody target of EGFR. It specifically binds to EGFR with an affinity that is about 5–10 times higher than that of the endogenous ligand and competitively blocks the endogenous EGFR ligand binding, thus suppressing the signal transmission of EGFR and causing a downregulation of the RAS–RAF–mitogen-activated protein kinase 1 (MAPK1) and the v-akt murine thymoma viral oncogene homolog 1 (AKT1) axis. This in turn inhibits the growth and migration of tumor cells (Zhao et al., 2017; Bray et al., 2019). A number of studies have shown that the clinical application of cetuximab reduces the overall mortality in colon cancer patients (Zhao et al., 2017). Although cetuximab is one of the currently successful tumor treatment drugs, a relatively high proportion of patients will eventually develop resistance to cetuximab, which subsequently leads to tumor recurrence and metastasis (Misale et al., 2014; Garcia-Foncillas et al., 2019). Approximately 40% of CRC patients harbor genetic disorders such as mutations in the v-Ki-ras2 Kirsten rat sarcoma viral oncogene homolog (KRAS), phosphatidylinositol-4,5-bisphosphate 3-kinase catalytic subunit alpha (PIK3CA), phosphatase and tensin homolog deleted on chromosome 10 (PTEN), and neuroblastoma-RAS (NRAS) in the EGFR pathway cascade. However, several patients without the above gene aberrations have initially responded to cetuximab therapy but later achieved acquired drug resistance. At present, the knowledge of acquired cetuximab resistance is still limited (Poehlmann et al., 2007; Raponi et al., 2008). Pumilio RNA-binding family member 1 (PUM1) is described as an oncogene that plays an important role in a variety of malignant tumors (Naudin et al., 2017; Guan et al., 2018; Dai et al., 2019), but its function in cetuximab resistance in CRC is unclear. In this study, we hypothesize that PUM1 promotes acquired cetuximab resistance in CRC. Thus, we established cetuximab-resistant colon cancer cell lines SW480WR and Caco-2R and investigated the mechanism of action of PUM1 in those cells.

## Materials and Methods

### Cell Culture and Establishment of Cetuximab-Resistance Cell Lines

The colon cancer cell lines SW480 (ATCC^®^ CCL-228^TM^) and Caco-2 (ATCC^®^ HTB-37^TM^) are commonly used in research. The cells are also sensitive to cetuximab and can be cultivated to cetuximab-resistant cell lines; thus, we selected the above cell lines as parent cells. Cells were cultured in Eagle’s minimum essential medium (EMEM, Catalog No. 30-2008) with 10–20% of fetal bovine serum (FBS, Gibco) and 1% of penicillin/streptomycin (Gibco, 15140-122). Cells were cultured in a 75-cm^2^ flask and maintained in an incubator at 37°C with 5% CO_2_. When cells achieved 70–80% confluence, they were trypsinized and passaged as 1:3 dilution. To generate the cetuximab-resistant cell lines, cetuximab was purchased from Merck (Darmstadt, Germany). First, we determined the cetuximab concentration causing 50% growth inhibition (IC50) of SW480 and Caco-2 cell lines by administering cetuximab for 10–14 days. Concurrently, the SW480 and Caco-2 cells were exposed to cetuximab at a concentration of IC50 and cultured the cells for 10 days, then we gradually increased the cetuximab concentration (2 μg/ml) every 10 days for 3 months. Eventually, the drug-resistant cells could survive at a concentration of cetuximab 10 times higher than the IC50. Resistant cells were tested to confirm the acquired resistance by reassessing the dose–response of cetuximab after 6 weeks in cell culture without cetuximab (Boeckx et al., 2015).

### Cell Proliferation Assay

Cell viability was determined by the Cell Counting Kit-8 (CCK-8, GK10001, GLPBIO) assay (Jiang et al., 2018). When the cells reached 70–80% confluence, they were harvested by trypsin, resuspended, and set up in a 96-well plate at 1,000/well. After 96 h, the viabilities of the cells were tested according to the manufacturer’s instruction.

### Immunoblot

Antibodies for PUM1 (PA5-30327) were purchased from Thermo Fisher Scientific, and those for DEAD-box helicase 5 (DDX5, #4387) were from Cell Signaling Technology. Antibody for β-actin (C4) (sc-47778) was purchased from Santa Cruz Biotechnology.

For immunoblot, cells were lysed on ice in 1× lysis buffer (Cat. no. 00-4333-57, Thermo) with 1× Protease Inhibitor Cocktail (Cat no. 78429, Thermo). Proteins were measured by Micro BCA Protein Assay Kit (Thermo, Prod#23235). Ten micrograms of total protein from each sample were resolved on 10% sodium dodecyl sulfate Bis-Tris HCl polyacrylamide gel with running buffer and transferred onto 0.2 μm polyvinylidene fluoride membranes (Bio-Rad, Hercules, CA, United States). The membranes were incubated in 5% non-fat milk on shaker for 1 h, followed by probing with first antibody of 2 h and incubated with corresponding second antibody for 1 h. Images were obtained using an enhanced chemiluminescence (ECL) system (Super Signal West Dura Kit, Thermo) (Yao et al., 2015).

### PUM1 and DDX5 Knockout and DDX5 Overexpression

Clustered regularly interspaced short palindromic repeats (CRISPR)-caspase 9 (Cas9) plasmids (C11005) carrying PUM1 or DDX5 sgRNAs were constructed by GenePharma (Shanghai, China). Two types of PUM1 and DDX5 sgRNAs were produced, named PUM1 sgRNA-1 and PUM1 sgRNA-2 and DDX5 sgRNA-1 and DDX5 sgRNA-2, respectively. We transfected the CRISPR-Cas9 plasmids carrying PUM1 or DDX5 to SW480R or Caco-2R cell lines with Lipofectamine 2000 (11668019, Thermo Fisher Scientific). The stably transfected cells were selected in the presence of 5 μg/ml puromycin for 2 weeks (Rahimi et al., 2019). DDX5 overexpression was also performed with pSIN-GFP carrying DDX5 coding sequence (CDS) without 3′ untranslated region (UTR) and transfected to corresponding colon cancer cell lines by Lipofectamine 2000 according to the manufacturer’s instruction. The stably transfected cells were selected in the presence of 5 μg/ml puromycin for 2 weeks.

### Quantitative Reverse Transcription PCR

Total RNA was extracted from SW480 or Caco-2 cells using AllPrep RNA/Protein kit (Cat. 80404, QIAGEN). cDNA synthesis was performed using iScript Reverse Transcription Supermix (#170-8841, Bio-Rad). Primers of DDX5 and glyceraldehyde 3-phosphate dehydrogenase (GAPDH) were ordered from Bio-Rad.

Quantitative reverse transcription PCR (qRT-PCR) for associated genes was performed using the SsoAdvanced Universal SYBR Green Supermix (#172-5124, Bio-Rad) and run for 40 cycles at 95°C for 2 min, then at 95°C for 5 s and at 60°C for 30 s. The temperature was increased by 0.5°C every 5 s, starting from 65°C until it reached 95°C. All assays followed the manufacturer’s protocol with 2^–ΔΔ*ct*^ method.

### mRNA Stability Assay

Actinomycin D (#15021) was purchased from Cell Signaling Technology. Cells were cultured and harvested with trypsin when the cells reached 70–80% confluence. Cells were washed with PBS and resuspended in a six-well plate (3 × 10^5^ cells per well). After the cells were attached, actinomycin D (1 mg/ml) was added, and cells were collected at 0, 2, 4, 6, and 8 h after addition of actinomycin D. The RNA of each well was extracted. mRNA expression level changes were detected by real-time fluorescent quantitative PCR (Kuwano et al., 2015).

### Dual-Luciferase Assays

We constructed the 3′ UTR of DDX5 into the plasmid psiCHECK2 (C8021, Promega) carrying double fluorescent reporter gene, then we transfected an empty plasmid or the constructed plasmid containing 3′ UTR sequence of DDX5 to cells. After 48 h of transfection, cells were collected and tested according to the manufacturer’s instructions (Bailey et al., 2015; Lu et al., 2017).

### RNA Pull-Down Assay

SW480R and Caco-2R cells were cultured to 70–80% confluence and transfected with biotin-labeled DDX5 5′ UTR, CDS, and 3′ UTR sequence. Biotin-labeled DDX5 5′ UTR, CDS, and 3′ UTR sequence were purchased from GenePharma (Shanghai, China). The cells were collected after 48 h and incubated with streptavidin magnetic beads (Pierce) overnight at 4°C. Cells were then lysed in lysis buffer with protease and phosphatase inhibitors and centrifuged to remove the liquid phase to obtain the protein-bound magnet beads. The protein bound on the magnetic beads was dissociated in RNA-binding buffer and eluted in Laemmli lysis buffer. The expressions of PUM1 and β-actin protein were then detected by western blot (Kuwano et al., 2015).

### Statistical Analysis

Data were presented as mean ± SD and were analyzed by one- or two-way ANOVA followed with a Dunnett’s *post hoc* test. The difference was considered statistically significant when *p*-value was less than 0.05.

## Results

### PUM1 Was Upregulated in Cetuximab-Resistant Colon Cancer Cells

**FIGURE 1:**
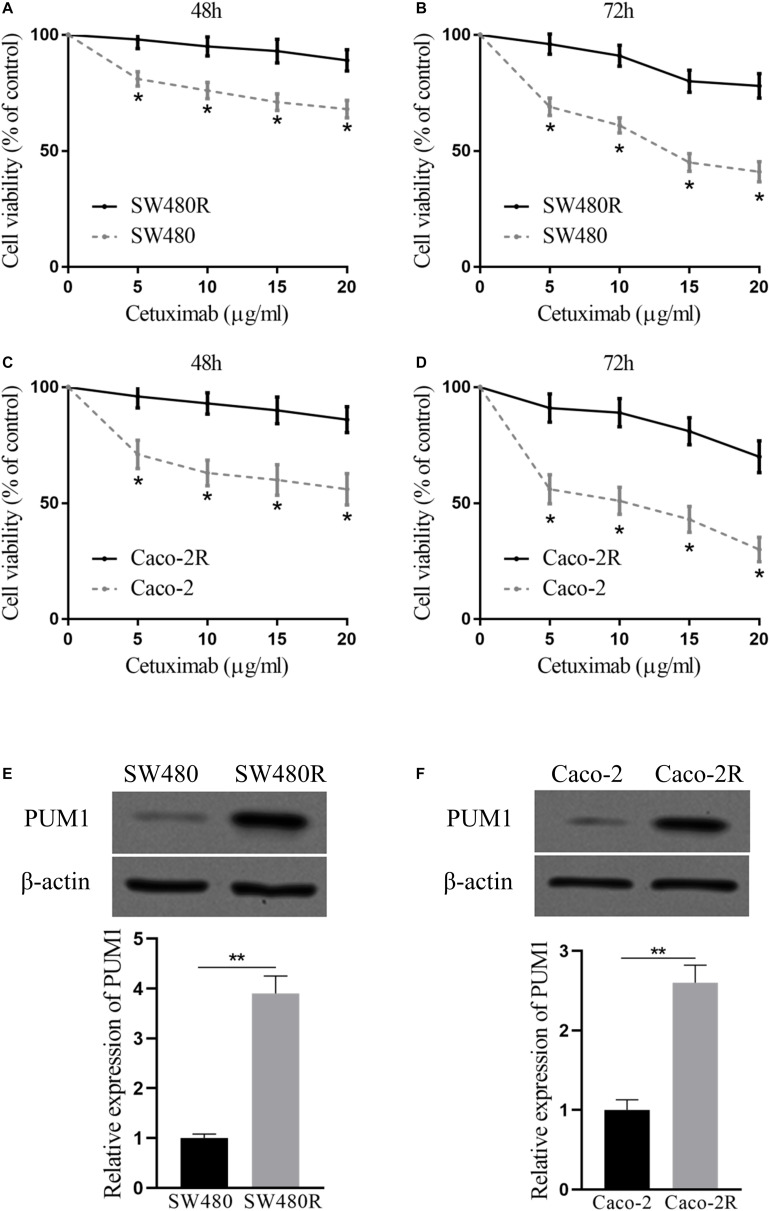
Pumilio RNA-binding family member 1 (PUM1) is upregulated in cetuximab-resistant colon cancer cells. SW480 and SW480R cells were incubated with cetuximab for **(A)** 48 h and **(B)** 72 h, and cell proliferation was determined using the Cell Counting Kit-8 (CCK-8) assay. Caco-2 and Caco-2R cells were incubated with cetuximab for **(C)** 48 h and **(D)** 72 h, and cell proliferation was determined using the CCK-8 assay. **(E,F)** Western blot analysis of PUM1 levels in colon cancer cells. β-actin served as a loading control. Data were presented as mean ± SD of three independent experiments. *p < 0.05, **p < 0.01.

We established two cetuximab-resistant colon cancer cell lines from SW480 and Caco-2, named SW480R and Caco-2R, respectively. To examine the resistance ability of the established cells, we incubated the cells with cetuximab in different concentrations (0–20 μg/ml) for 48 and 72 h and determined cell proliferation with the CCK-8 assay. The concentration range (0–20 μg/ml) of cetuximab can greatly inhibit the parent cells but has little effect on the drug-resistant cells. The viability of the parent cells of SW480 decreased from 85 to 75%, whereas the viability of SW480R decreased only by 3% at the maximal concentration of cetuximab (20 μg/ml). The differences between the SW480 and SW480R cells were clear in every concentration of cetuximab (Figure 1A). The differences in cell viability were greater (48% of SW480 and 92.5% of SW480R) in 72 h of incubation with cetuximab (Figure 1B). Similar results were also observed in Caco-2 and Caco-2R cells (Figures 1C,D). Next, we checked the expressions of PUM1 in parent cells and cetuximab-resistant cells with immunoblot. Results indicate that PUM1 was more strongly expressed in SW480R and Caco-2R cells compared with the parent cells (Figures 1E,F). These results indicate that PUM1 is upregulated in cetuximab-resistant colon cancer cell lines SW480 and Caco-2.

### Suppression of PUM1 Inhibited Proliferation of Cetuximab-Resistant Colon Cancer Cells

**FIGURE 2:**
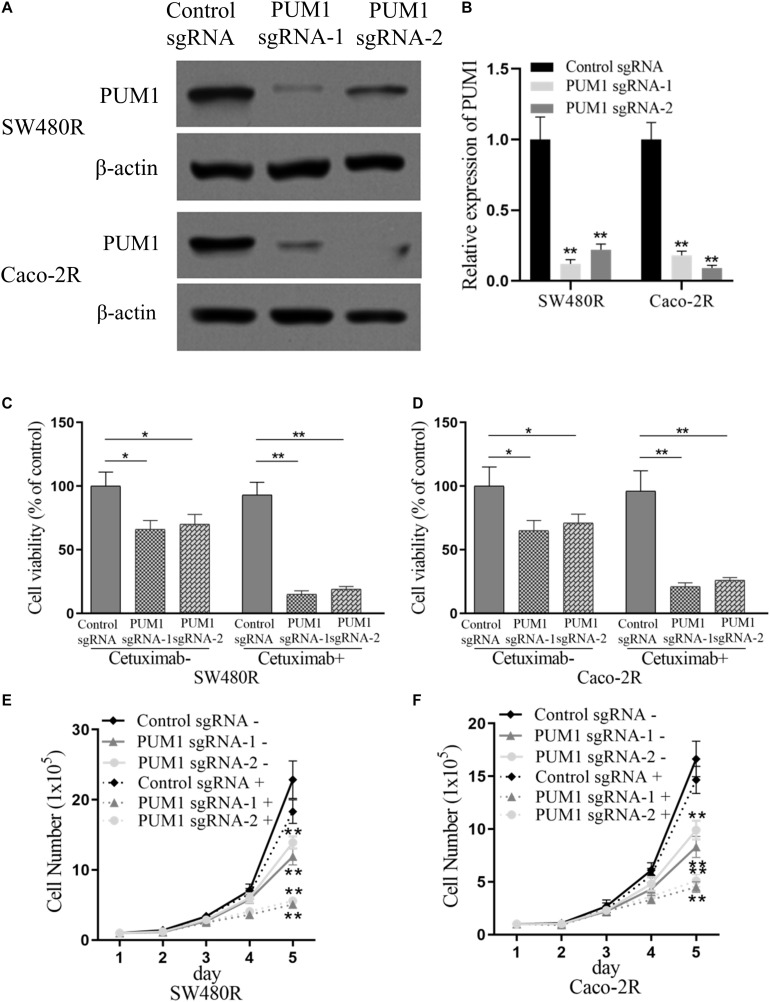
Suppression of pumilio RNA-binding family member 1 (PUM1) inhibits proliferation of cetuximab-resistant colon cancer cells. **(A,B)** Western blotting assay for the levels of PUM1 in SW480R and Caco-2 colon cancer cells. β-actin served as a loading control. **(C)** SW480R and **(D)** Caco-2R cells were incubated with or without cetuximab for 72 h, and cell proliferation was determined using the Cell Counting Kit-8 (CCK-8) assay. **(E)** SW480R and **(F)** Caco-2R cells were grown in six-well plates, and the cell number was determined. Cetuximab+ and cetuximab– represent the culture media with and without 10 μg/ml cetuximab, respectively. Data were presented as mean ± SD of three independent experiments. *p < 0.05; **p < 0.01.

To further investigate PUM1’s influence on the proliferation of drug-resistant colon cancer cells treated with cetuximab, we performed PUM1 knockout experiments using CRISPR-Cas9 carrying PUM1 sgRNA. We tested two types of sgRNAs named PUM1 sgRNA-1 and PUM1 sgRNA-2. Figures 2A,B show the successful PUM1 knockout in both SW480R and Caco-2R cells. Then, we checked those PUM1 knockout cells with or without cetuximab for 72 h. The cell viabilities were impaired by about 25–30% by PUM1 knockout in SW480R cells without cetuximab, which means PUM1 played a role in promoting cell proliferation. When cetuximab was added in cell culture media, the cell viability of PUM1 sgRNA-1 and PUM1 sgRNA-2 cells was impaired by about 80–85% compared with their control sgRNA SW480 cells (Figure 2C). The Caco-2R cell line exhibited similar results (Figure 2D). We counted the living cell numbers of those conditional cells (Figures 2E,F) cultured with or without cetuximab and observed a trend consistent with that of the cell proliferation assay. The above experiments indicate that knockdown of PUM1 inhibited the proliferation of cetuximab-resistant colon cancer cells of SW480 and Caco-2. Moreover, the cetuximab-resistant colon cancer cells lost their resistance to their corresponding drug.

### PUM1 Positively Regulated DDX5 mRNA Expression Through DDX5 3′ UTR

**FIGURE 3:**
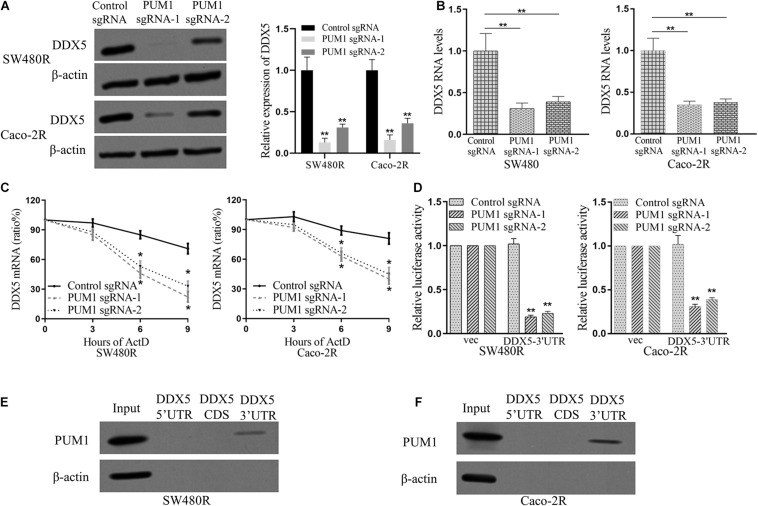
Pumilio RNA-binding family member 1 (PUM1) positively regulates DEAD-box helicase 5 (DDX5) mRNA expression through DDX5 3′ untranslated region (UTR). **(A)** Western blotting analysis of DDX5 proteins in both PUM1-deleted SW480R and Caco-2R cells. **(B)** β-actin was used as a loading control. Relative quantities of DDX5 mRNAs, extracted from sgRNA-control and PUM1-KO cells, were estimated using qRT-PCR. **(C)** Glyceraldehyde 3-phosphate dehydrogenase (GAPDH) serves as a control. The mRNA decay rate of DDX5 as indicated in SW480R and Caco-2R cells by qRT-PCR after suppressing PUM1 expression followed by actinomycin D treatment (10 mg/ml) for specified time points. **(D)** GAPDH serves as a control. Dual-luciferase assays showing the luciferase of DDX5 3′ UTR following PUM1-KO. The empty 3′ UTR luciferase plasmid served as a loading control. Synthetic RNA oligos with the indicated PUM1 consensus sequences for DDX5 were examined through RNA pull-down and blotted for PUM1 in **(E)** SW480R and **(F)** Caco-2R cells. Data are mean ± SD of three independent experiments, and each was measured in triplicate. *p < 0.05, **p < 0.01.

Studies have described that PUM1 and DDX5 promote CRC (Sarkar et al., 2015; Naudin et al., 2017; Guan et al., 2018; Dai et al., 2019). To test our hypothesis whether DDX5 was a target regulated by PUM1 in cetuximab-resistant cells, we performed immunoblot to examine the expression of DDX5 under the condition of PUM1 knockout. The expression of DDX5 significantly decreased following the silencing of PUM1 in both sgRNA-1 and sgRNA-2 of SW480R and Caco-2R cells (Figure 3A). mRNA expressions of DDX5 also decreased (as determined by qRT-PCR in those cells) (Figure 3B). Next, we checked the DDX5 mRNA stability and mRNA decay by adding actinomycin D to inhibit its transcription. At time points 0, 3, 6, and 9 h with actinomycin D, cells were harvested, mRNAs were extracted, and the mRNA expression levels were detected by real-time fluorescent quantitative PCR. The results indicate that both PUM1 sgRNA-1 and PUM1 sgRNA-2 were inhibited greatly by actinomycin D compared with their control sgRNAs, which meant that they were mature sgRNAs performing sgRNA functions (Figure 3C). We then amplified the fragments of 3′ UTR region of DDX5 by PCR and inserted the fragments to plasmid psiCHECK2 and transfected into PUM1 knockout SW480R and Caco-2R cells. We observed that 75–80% of luciferase activities were suppressed in PUM1 sgRNA-1 and PUM1 sgRNA-2 SW480R cells compared with control sgRNA. Similarly, Caco-2R cells demolished about 60–70% of luciferase activities (Figure 3D). The experiments of RNA pull-down further proved PUM1 interacted with DDX5 in the region of 3′ UTR, rather than 5′ UTR or CDS (Figures 3E,F). These results clearly prove that PUM1 positively regulates DDX5 by interacting with its 3′ UTR.

### Suppression of DDX5 Inhibited the Proliferation of Cetuximab-Resistant Colon Cancer Cells

**FIGURE 4:**
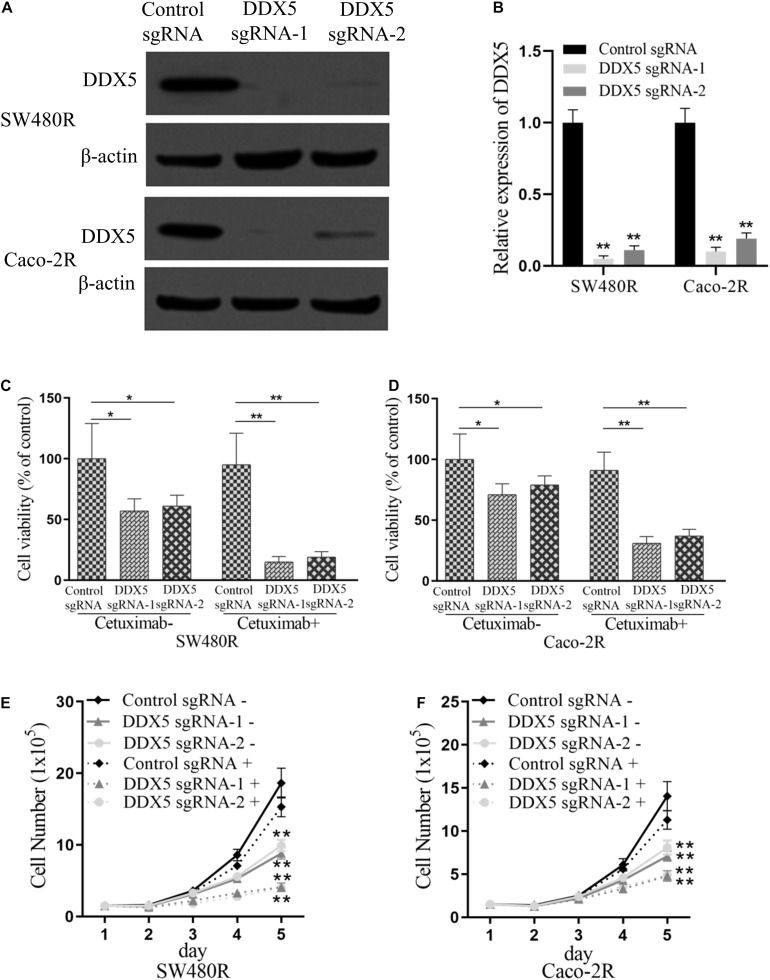
Suppression of DEAD-box helicase 5 (DDX5) inhibits the proliferation of cetuximab-resistant colon cancer cells. Western blotting assay for the levels of DDX5 in **(A)** SW480R and **(B)** Caco-2R colon cancer cells. β-actin served as a loading control. **(C)** SW480R and **(D)** Caco-2R cells were incubated with or without cetuximab for 72 h, and cell proliferation was determined using the Cell Counting Kit-8 (CCK-8) assay. **(E)** SW480R and **(F)** Caco-2R cells were grown in six-well plates, and the cell number was determined. Cetuximab+ and cetuximab– represent the culture media with and without 10 μg/ml cetuximab, respectively. Data were presented as mean ± SD of three independent experiments. *p < 0.05; **p < 0.01.

To further elucidate the role played by DDX5 in cetuximab resistance in colon cancer cells, we performed DDX5 knockdown in SW480R and Caco-2R cells with plasmid CRISPR-Cas9 carrying DDX5 sgRNA-1 and sgRNA-2 (Figures 4A,B). Cell proliferation was checked by the CCK-8 assay. As expected, the cell viabilities of DDX5 knockout exhibited lower percentages (35–40% lower) compared to their controls. With the addition of cetuximab, the cell viabilities were significantly lower (by 75–80%) than their controls in both sgRNA-1 and sgRNA-2 of SW480R cells (Figure 4C). Similar results were observed in Caco-2R cells (Figure 4D). Living cell number count was consistent with the corresponding cell viability in each cell graph (Figures 4E,F). These data show that the suppression of DDX5 inhibited the proliferation of cetuximab-resistant colon cancer cells SW480R and Caco-2R.

### DDX5 Was Critical for PUM1-Mediated Cetuximab Resistance

**FIGURE 5:**
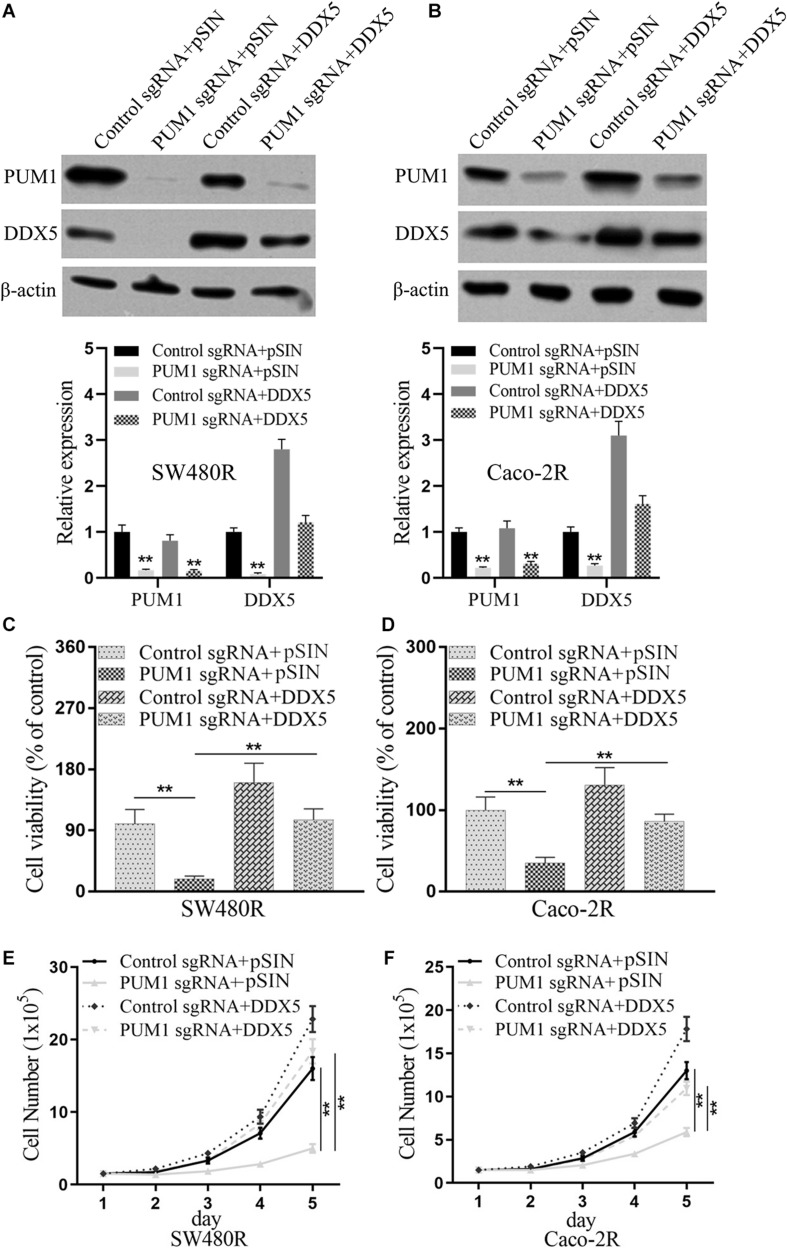
DEAD-box helicase 5 (DDX5) is critical for pumilio RNA-binding family member 1 (PUM1)-mediated cetuximab resistance. Western blotting assay for the levels of PUM1 in **(A)** SW480R and **(B)** Caco-2 colon cancer cells. β-actin served as a loading control. **(C)** SW480R and **(D)** Caco-2R cells were incubated with or without cetuximab for 72 h, and cell viability was determined using the Cell Counting Kit-8 (CCK-8) assay. **(E)** SW480R and **(F)** Caco-2R cells were grown in six-well plates, and the cell number was determined. Data were presented as mean ± SD of three independent experiments. **p < 0.01.

To study the role of regulation of PUM1 on DDX5 in cetuximab-resistant cells, we performed co-transfection with PUM1 knockdown and DDX5 overexpression in SW480R and Caco-2R cells. Results are shown in Figures 5A,B. When co-transfected with control sgRNA and empty vector (pSIN) to SW480R cells, the expressions of PUM1 and DDX5 provided us a baseline (Figure 5A, first lane); when we knocked out PUM1 with its sgRNA, the expression of DDX5 subsequently decreased, which means PUM1 positively regulated DDX5 downstream (Figure 5A, second lane). We then performed the DDX5 overexpression plus control sgRNA, results of which showed that PUM1 was expressed at the standard level and DDX5 was expressed at a stronger level compared with the first lane (Figure 5A, third lane). However, when we co-transfected PUM1 sgRNA with DDX5 overexpression, the expression level of DDX5 was alleviated by PUM1 knockdown (Figure 5A, fourth lane). The housekeeping protein β-actin presented the same amount of protein loading (Figure 5A). The Caco-2R cells showed similar results as SW480R did (Figure 5B). These experiments clearly demonstrated that PUM1 positively regulated DDX5 in cetuximab-resistant colon cancer cells. When we examined the cell viabilities with those transfectants, results reveal that PUM1 knockout impaired cell proliferation but DDX5 overexpression promoted it. PUM1 knockout could suppress this effect of DDX5 to cell proliferation in SW480R cells (Figure 5C). The cell number count provided further consistent results with cell viability experiments (Figure 5E). The Caco-2R cells presented similar trends as well (Figures 5D,F). The above data prove that PUM1 positively regulated DDX5, and DDX5 was a critical factor involved in PUM1-mediated cetuximab resistance.

## Discussion

Cetuximab is an effective EGFR antibody that was proven to decrease the overall mortality rate in CRC patients. However, the acquired resistance to this drug causes tumor recurrence and metastasis (Misale et al., 2014; Zhao et al., 2017; Garcia-Foncillas et al., 2019). The mechanisms of cetuximab resistance were studied extensively. Some studies described EGFR mutations (Bagchi et al., 2018; Tintelnot et al., 2019) or downregulation of signal pathway mutations including KRAS-MAPK (Diaz et al., 2012; Misale et al., 2012), phosphoinositide 3-kinase (PI3K)-PTEN-AKT (Goel et al., 2004), and Janus kinase (JAK)/signal transducer and activator of transcription (STAT) as causes of cetuximab resistance (Zhao et al., 2017). In addition, abnormal regulation of miRNAs (Lu et al., 2017) and several proteins (Leonard et al., 2018) has also been revealed to contribute to cetuximab resistance in CRC. However, there are a number of patients who developed chemoresistance to cetuximab without gene alterations as mentioned in previous studies. As a result, more and more candidates targeting cetuximab resistance were discovered and described.

PUM1 belongs to a member of pumilio RNA-binding protein family. It was recently discovered to play a role in the development of various cancers (Lee et al., 2007, 2016; Naudin et al., 2017; Guan et al., 2019), such as ovarian cancer (Guan et al., 2018, 2019), pancreatic cancer (Dai et al., 2019), and leukemia (Naudin et al., 2017), in which PUM1 acted as a tumor promoter. In this study, we found that PUM1 was upregulated in the established cetuximab-resistant colon cancer cell lines SW480R and Caco-2R compared with their parent cell lines (Figure 1), which implies that PUM1 is involved in promoting acquired cetuximab resistance. Then, we knocked out PUM1 using its sgRNAs in SW480R and Caco-2R cells and found that the proliferation of PUM1-silenced cells was greatly suppressed (Figure 2). To further investigate the downstream signals that PUM1 regulated, we focused on DDX5, a member of the DEAD-box RNA helicase family P68. DDX5 is considered an oncogene in a number of cancers. It is overexpressed in the most common solid tumors, that is, breast, prostate, and colon (Janknecht, 2010). As a result, DDX5 became a new target for cancer treatment. In our study, we found that PUM1 interacted with DDX5 with its 3′ UTR and positively regulated it in SW480R and Caco-2R cells (Figure 3). Suppression of DDX5 inhibited the proliferation of cetuximab-resistant colon cancer cells (Figure 4). Our results provide evidence that DDX5 is positively regulated by PUM1. Finally, we co-transfected PUM1 sgRNAs and DDX5 overexpression vectors to SW480R and Caco-2R cells, the results of which exhibited that the overexpression of DDX5 could be relieved by PUM1 silencing (Figure 5), suggesting that DDX5 is critical in PUM1-mediated regulation in cetuximab-resistant colon cancer cells. Our study is the first to provide evidence that PUM1 played an important role in cetuximab resistance, and DDX5 is critical for PUM1-mediated cetuximab resistance in CRC. In this study, we have not examined whether acquired mutation occurred in SW480R or Caco-2R cells nor have we investigated the potential factor PUM1 or DDX5 interacting within the downstream signal pathways of EGFR. Patient samples with/without cetuximab resistance need to be collected as well. Further studies need to be performed to understand the detailed mechanisms of cetuximab resistance and provide the clinical significance in preventing cetuximab resistance of CRC by inhibiting PUM1.

## Conclusion

In conclusion, our study provided a new candidate treatment target, PUM1, which plays an important role in cetuximab resistance. Suppression of PUM1 may be a useful target to overcome cetuximab resistance in CRC. Our study provides new insight into future research in preventing cetuximab resistance of CRC by inhibiting PUM1.

## Data Availability Statement

The raw data supporting the conclusions of this article will be made available by the authors, without undue reservation.

## Author Contributions

QL, CX, YKC, JY, YYC, WZ, and LY conceived and designed the study and wrote the manuscript. QL, WZ, and LY studied the supervision, coordination, funding support, design of this study, drafting of the article, and final approval of the version to be published.

## Conflict of Interest

The authors declare that the research was conducted in the absence of any commercial or financial relationships that could be construed as a potential conflict of interest.

## Publisher’s Note

All claims expressed in this article are solely those of the authors and do not necessarily represent those of their affiliated organizations, or those of the publisher, the editors and the reviewers. Any product that may be evaluated in this article, or claim that may be made by its manufacturer, is not guaranteed or endorsed by the publisher.
